# Glycemic Variability and Its Association with Traditional Glycemic Control Biomarkers in Patients with Type 1 Diabetes: A Cross-Sectional, Multicenter Study

**DOI:** 10.3390/jcm14072434

**Published:** 2025-04-02

**Authors:** Sandra Lazar, Delia-Viola Reurean-Pintilei, Ioana Ionita, Vlad-Florian Avram, Andreea Herascu, Bogdan Timar

**Affiliations:** 1 Doctoral School of Medicine, “Victor Babes” University of Medicine and Pharmacy, 300041 Timisoara, Romania; sandra.lazar@umft.ro (S.L.); andreea.herascu@umft.ro (A.H.); 2 First Department of Internal Medicine, “Victor Babes” University of Medicine and Pharmacy, 300041 Timisoara, Romania; ionita.ioana@umft.ro; 3 Department of Hematology, Emergency Municipal Hospital, 300254 Timisoara, Romania; 4 Centre for Molecular Research in Nephrology and Vascular Disease, “Victor Babes” University of Medicine and Pharmacy, 300041 Timisoara, Romania; avram.vlad@umft.ro (V.-F.A.); bogdan.timar@umft.ro (B.T.); 5 Department of Medical-Surgical and Complementary Sciences, Faculty of Medicine and Biological Sciences, “Stefan cel Mare” University, 720229 Suceava, Romania; 6 Consultmed Medical Centre, Department of Diabetes, Nutrition and Metabolic Diseases, 700544 Iasi, Romania; 7 Multidisciplinary Research Center for Malignant Hematological Diseases (CCMHM), Victor Babes University of Medicine and Pharmacy, 300041 Timisoara, Romania; 8 Second Department of Internal Medicine, “Victor Babes” University of Medicine and Pharmacy, 300041 Timisoara, Romania; 9 Department of Diabetes, “Pius Brinzeu” Emergency Hospital, 300723 Timisoara, Romania

**Keywords:** glycemic variability, glycemic control, hemoglobin A1c, time in range, standard deviation

## Abstract

**Background/Objectives:** Glycemic variability (GV) is a novel concept in the assessment of the quality of glycemic control in patients with diabetes, with its importance emphasized in patients with type 1 diabetes. Its adoption in clinical practice emerged with the increased availability of continuous glycemic monitoring systems. The aim of this study is to evaluate the GV in patients with type 1 diabetes mellitus (T1DM) and to assess its associations with other parameters used to evaluate the glycemic control. **Methods:** GV indexes and classical glycemic control markers were analyzed for 147 adult patients with T1DM in a multicentric cross-sectional study. **Results:** Stable glycemia was associated with a higher time in range (TIR) (78% vs. 63%; *p* < 0.001) and a lower HbA1c (6.8% vs. 7.1%; *p* = 0.006). The coefficient of variation (CV) was reversely correlated with TIR (Spearman’s r = −0.513; *p* < 0.001) and positively correlated with hemoglobin A1c (HbA1c) (Spearman’s r = 0.349; *p* < 0.001), while TIR was reversely correlated with HbA1c (Spearman’s r = −0.637; *p* < 0.001). The composite GV and metabolic outcome was achieved by 28.6% of the patients. **Conclusions:** Stable glycemia was associated with a lower HbA1c, average and SD of blood glucose, and a higher TIR. A TIR higher than 70% was associated with a lower HbA1c, and SD and average blood glucose. Only 28.6% of the patients with T1DM achieved the composite GV and metabolic outcome, despite 53.7% of them achieving the HbA1c target, emphasizing thus the role of GV in the assessment of the glycemic control.

## 1. Introduction

Type 1 diabetes mellitus (T1DM) is a chronic, complex metabolic disorder characterized by the destruction of insulin-producing pancreatic beta cells. This disruption in the body’s ability to produce its own insulin leads to an inability to properly regulate blood glucose (BG) levels, a hallmark feature of this autoimmune disease [1].

Due to the underlying pathological mechanism of T1DM, which results in absolute insulin deficiency, individuals with T1DM experience wide and unpredictable fluctuations in their BG levels. These glycemic excursions can result in potentially dangerous dysglycemic episodes [2]. Moreover, this lack of glycemic control can ultimately contribute to the development of various microvascular and macrovascular complications, such as neuropathy, nephropathy, retinopathy, and cardiovascular disease (CVD) [3,4].

Epidemiological studies have shown individuals with T1DM have a 4- to 10-fold higher risk of CVD compared to the general population. Even moderately elevated HbA1c levels can significantly increase the risk of cardiovascular complications in this patient group [5,6,7].

Regarding diabetic retinopathy, studies have consistently reported a high prevalence—around 80%—of any stage of the condition in patients with T1DM after more than five years of disease progression, with it being considered one of the leading causes of preventable blindness. However, the landmark Diabetes Control and Complications Trial demonstrated that proper diabetes management can reduce the risk of retinopathy progression by more than 50% [8,9].

Similarly, for diabetic nephropathy, 20 to 50% of T1DM patients with unstable BG control may develop kidney complications within 20 years of disease progression [10,11,12]. Diabetic neuropathy has been reported to have an incidence rate of 20% to 40% in individuals with T1DM. Furthermore, research has shown that maintaining proper blood glucose control is one of the most crucial strategies for preventing and managing diabetic neuropathy [13,14,15].

The burden of managing these complications can have a profound impact, not only on the individual’s physical health and overall quality of life but also on their emotional wellbeing and psychological functioning. Moreover, the significant financial costs associated with the management of these complications pose a substantial burden on healthcare systems, potentially straining resources and reducing the availability of care for other patients. Additionally, the reduced workforce productivity stemming from the challenges of living with diabetes can have far-reaching economic implications, affecting both the individual and the broader community [16,17].

An optimal diabetes management approach, consisting of lifestyle changes such as a healthy diet and regular physical activity, as well as effective BG management through proper insulin administration and close monitoring, can significantly reduce the risk, appearance, and development of these debilitating diabetes-related complications [18,19].

For a long period of time, one of the most widely used tools for quantifying glycemic control was the glycated hemoglobin (HbA1c) level [20]. However, HbA1c alone does not provide a complete picture of an individual’s glycemic control as it is unable to capture the day-to-day or even hour-to-hour fluctuations in BG levels, a phenomenon known as glycemic variability (GV) [21].

GV refers to oscillations in BG levels and encompasses both short- and long-term variations, including the amplitude, frequency, and speed at which BG levels rise or fall [22].

Prior to the development of continuous glucose monitoring systems (CGMSs), assessing GV was a significant challenge. The availability of these advanced technologies in clinical practice has led to the widespread adoption of GV as a complementary and complex indicator for evaluating the quality of glycemic control [23]. Emerging evidence has firmly established GV as a reliable predictor of the development and progression of diabetes-related complications even in individuals with well-controlled HbA1c levels. This relationship is particularly concerning as GV has been linked to an increased risk of adverse clinical outcomes, including severe hyperglycemia and clinically significant hypoglycemia, both being associated with an elevated risk of CVD [24,25].

Current literature data indicate that higher GV may be more harmful than chronic hyperglycemia alone [26,27]. It was reported that GV causes greater oxidative stress, inflammation, and endothelial dysfunction, which can accelerate vascular complications. While chronic hyperglycemia contributes to long-term damage through advanced glycation end-products and metabolic stress, GV triggers acute and repeated episodes of oxidative stress due to rapid glucose fluctuations [28]. This can lead to excessive mitochondrial reactive oxygen species production, overwhelming the body’s antioxidant defenses more than sustained high glucose levels. Additionally, GV provokes stronger inflammatory responses, resulting in greater endothelial dysfunction and vascular injury. Studies show that intermittent hyperglycemia has a more profound effect on endothelial cell apoptosis and nitric oxide depletion compared to stable hyperglycemia, leading to impaired vasodilation and increased cardiovascular risk (CVR) [29,30,31,32]. Furthermore, GV is associated with heightened autonomic dysfunction, increasing the likelihood of arrhythmias and cardiovascular events (CVEs). Given these mechanisms, GV not only exacerbates diabetes-related complications but also presents an independent risk factor for CVD, making its management a critical priority in diabetes care [24].

Therefore, by quantifying the degree of fluctuations in BG levels, GV can provide healthcare professionals with a valuable tool to guide more effective treatment strategies and improve overall patient outcomes [33,34,35].

There are several key metrics used to evaluate GV through CGMSs. These includes time in range (TIR), which reflects the percentage of time a person’s BG levels remain within a target range, and metrics used to evaluate the spread or dispersion of glycemic values, such as standard deviation (SD) and coefficient of variability (CV), that provide insight into the magnitude of glycemic fluctuations [36].

CV is considered a more reliable parameter than SD for assessing GV and it represents the ratio of the SD to the mean glucose value, expressed as a percentage. This metric considers both the magnitude and the scale of glycemic values, providing a standardized and meaningful measure of GV [36].

Current clinical guidelines recommend for T1DM patients to achieve a CV below 36% and a TIR at least 70% alongside the classical lower than 7% HbA1c, leading thus to the emergence of the need to define a new composite diabetes management target, as defined by the achievement of all three sub-components: HbA1c lower than 7% in parallel with a CV below 36% and a TIR higher than 70% [37].

Since the phenomenon of GV is more pronounced in patients with T1DM compared to type 2 diabetes mellitus (T2DM) patients, as individuals with T1DM do not benefit from the stabilizing effect provided by residual insulin secretion [38], the clinical impact of GV is emphasized in patients with T1DM. However, the role of GV beyond traditional markers remains insufficiently explored. The extent to which GV correlates with conventional biomarkers, and how these relationships may vary across real-world patients, is not yet well-established. Addressing these important knowledge gaps is crucial to improving risk stratification and refining treatment strategies specifically for individuals living with T1DM. Although several studies have established associations between GV and glycemic control, few have systematically examined these relationships in real-world, multicentric cohorts using composite targets of control (CV, TIR, HbA1c). Moreover, the frequency with which patients meet all three targets simultaneously, and the factors that predict GV and TIR achievements, remain poorly characterized. This study seeks to address these knowledge gaps.

Considering these premises, the aims of this study are to evaluate the GV phenomena in patients with T1DM, to assess the interaction between the magnitude of GV and the traditional biomarkers of glycemic control, and to identify which factors are associated with increased GV in patients with T1DM.

## 2. Materials and Methods

### 2.1. Study Design and Patients

In a cross-sectional, multicentric study design, 147 patients with T1DM, who were users of CGMSs for at least 6 months, were enrolled according to a consecutive-case population-based enrollment principle. All participants attended outpatient visits for the management of diabetes at Diabetes Centre of “Pius Brinzeu” Emergency Hospital, Timisoara (105 patients), or Consultmed Clinic, Iasi, Romania (42 patients). The enrolled patients were treated with insulin via basal bolus regimens (84 patients), consisting of basal insulin analogs (Toujeo, Lantus, or Tresiba) and prandial insulin analogs (Novorapid, Humalog, Fiasp, or Lyumjev) or insulin delivered via continuous insulin delivery systems (63 users of insulin pumps).

The study population comprised patients ranging from 18 to 75 years-of-age and it was conducted during 2023–2024. The study design did not incorporate any proactive screening for diabetes-related complications.

The study was conducted according to the principles of the Declaration of Helsinki. All patients provided signed written informed consent prior to their enrollment in the study. The study protocol and informed consent was approved by the Ethics Committee of the “Victor Babes” University of Medicine and Pharmacy, Timisoara, Romania (No. 50 from 19th October 2021).

The studied group’s baseline characteristics are presented in Table 1.

### 2.2. Anthropometrical, Clinical and Laboratory Assessments

In all patients, data regarding their age, gender, diabetes duration, and type of therapy was collected from the medical records. HbA1c was measured on the day of the visit, in a fasting state, using a traceable and DCCT-standardized chemiluminescence method. HbA1c is reported in the study as the percentage of A1c hemoglobin from the total hemoglobin and provides a weighted average of the glycemia in the last three months.

During the visit, height and weight were measured using calibrated stadiometers and body weight scales, and the body mass index was calculated by dividing the patient’s weight (measured in kilograms) by the square of patient’s height (measured in meters).

### 2.3. Glycemic Variability Assessment

All patients were users of CGMSs manufactured by Medtronic, either the Guardian 3 or Guardian Link sensors, using the same type of sensor, which were officially provided by the manufacturer. These CGMS devices automatically transmit glucose measurements to a receiver (that can be the insulin pump or a telephone device) every 5 min, without the need for manual scanning.

The evaluated timeframe was a period of 90 days prior to the moment of the visit. Patients who used the systems for less than 70% of the total designed timeframe for the study were not enrolled. The system was calibrated using self-monitoring blood glucose measurements using approved glucose meters in order to increase the assessment’s accuracy and to minimize possible errors. The sensor’s software delivered reports of the SD and CV of glucose values, and these were collected for the designed timeframe. At the same time, TIR, time above range (TAR), and time below range (TBR) were collected from the continuous glucose monitoring reports. The current diabetes management guidelines recommend a CV of less than 36%, this threshold being associated with stable glycemia, and recommend that a patient should have glycemia between 70 mg/dL and 180 mg/dL for more than 70% of the total time [37]. HbA1c was measured using the DCCT-standardized chemiluminescence method, which is a highly accurate and precise technique that has been widely adopted for HbA1c quantification.

### 2.4. Statistical Analysis

Data were collected and analyzed using the Statistical Package for Social Sciences (IBM Corp. Released 2023. IBM SPSS Statistics for Windows, Version 29.0.2.0 Armonk, NY, USA: IBM Corp). The results are presented as central tendency and dispersion indicators. Continuous variable’s distribution was tested for normality using Shapiro and Wilk’s test. A *p*-value higher than 0.05 was associated with non-significant differences in the variables from Gaussian distribution.

Continuous variables with Gaussian distribution are presented as average ± standard deviation and the statistical significance of the differences was assessed using unpaired Student’s *t*-tests (two means) and ANOVA tests (more than two means). For continuous variables with non-parametric distribution, the results are presented as median and [interquartile distance]—as described by the difference between the value at which the fourth quartile starts and the value at which the second quartile starts. The statistical significance of the differences between medians of non-parametric variables was assessed using Mann–Whitney U tests (two medians) and Kruskal–Wallis tests (more than two variables). In the case of multiple comparisons, to account for multiple comparisons and control for the risk of type I error we applied the Bonferroni correction. Adjusted *p*-values were calculated by multiplying the original *p*-values by the number of comparisons. A Bonferroni-adjusted *p*-value < 0.05 was considered statistically significant. Both Bonferroni-adjusted as well as raw *p*-values are presented.

To evaluate the strength of the association between two continuous variables, Spearman’s correlation coefficient was used.

To evaluate the impact of a continuous variable as an independent variable on a dichotomous variable (dependent variable), logistic regression models were built. In these models, the exponent of the slope coefficient was considered to be the odds ratio increase associated with a one-step increase in the independent variable. To build the model, predictors were added in the initial iteration based on their clinical relevance for the outcome. The inclusion in the final model was performed according to a stepwise backward algorithm, with the predictors being included in the model when the corresponding *p*-value was lower than 0.05 and excluded from the model when *p* was higher than 0.15.

The sample size was estimated considering the primary outcome for differences in TIR between patients with vs. those without stable glycemia. The sample size was calculated with MedCalc v. 22 to provide a statistical power of 80% with a confidence level of 95%. According to the estimation, presuming a 1:1 ratio between the groups, a sample size of 140 individuals was required.

In this study, the statistical significance threshold was considered a *p*-value lower than 0.05.

## 3. Results

**Figure 1:**
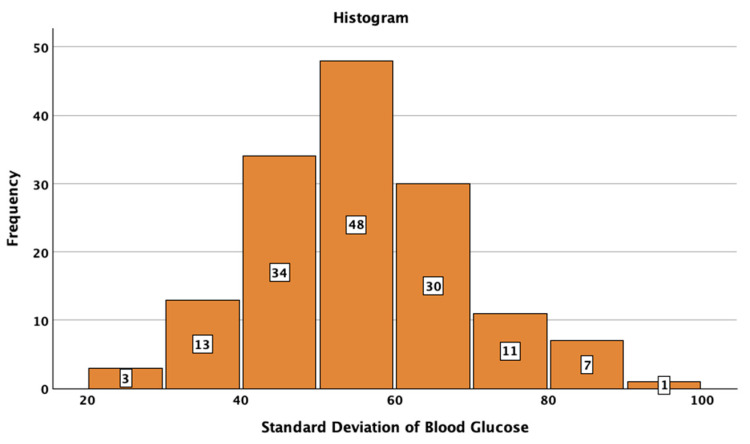
Histogram of standard deviation of blood glucose in the studied sample.

**Figure 2:**
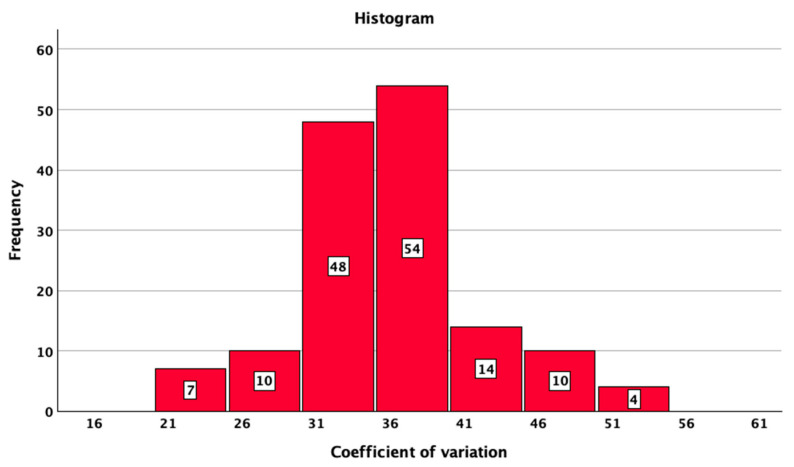
Histogram of coefficient of variation of blood glucose in the studied sample.

**Figure 3:**
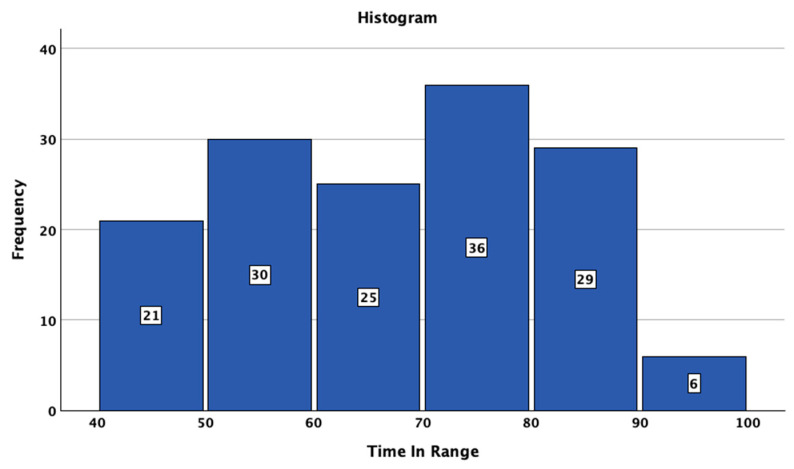
Histogram of time in range in the studied sample.

**Figure 4:**
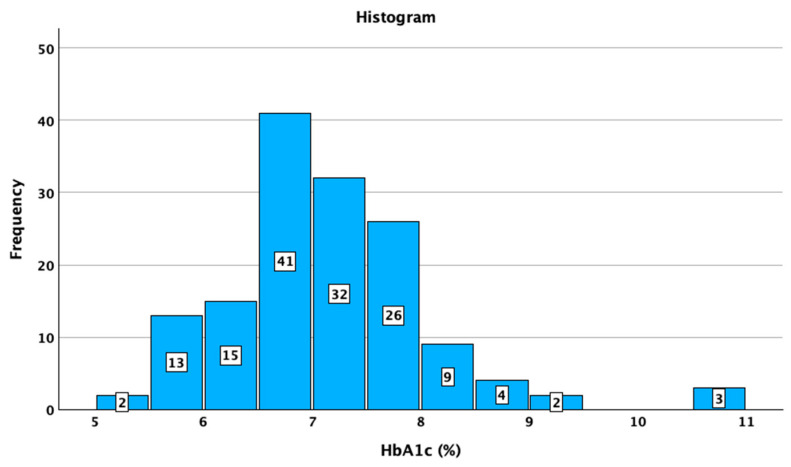
Distribution of HbA1c in the studied sample.

The studied patients with T1DM had a median SD of BG of 57 mg/dL (95%CI: 56–59), a median CV of 35.9 (95%CI: 34.5–37.0), and a median TIR of 69% 95%CI: 66–72%). The median HbA1c value was 7.0% (minimum 5.4%; maximum 10.5%). The distributions of SD, CV TIR, and HbA1c are presented in Figure 1, Figure 2, Figure 3 and Figure 4.

The detailed central tendency and dispersion indicators for GV indexes and HbA1c are presented in Table 2.

In the analyzed cohort, 45.6% of the patients achieved the recommended TIR of more than 70% of the monitored time, while 50.3% of the patients had stable glycemic values, as described by the achievement of a CV of BG values lower than 0.36. Regarding the HbA1c values, 53.7% of the patients reached the therapeutic target of HbA1c < 7%.

We proposed a composite GV and metabolic outcome, defined as achieving at the same time a TIR > 70%, CV < 0.36, as well as a HbA1c < 7%. This composite outcome was achieved by 28.6% of the patients, while 17.0% achieved two components of the composite outcome, 29.9% achieved one component of the outcome, and 24.5% achieved none of the components.

**Figure 5:**
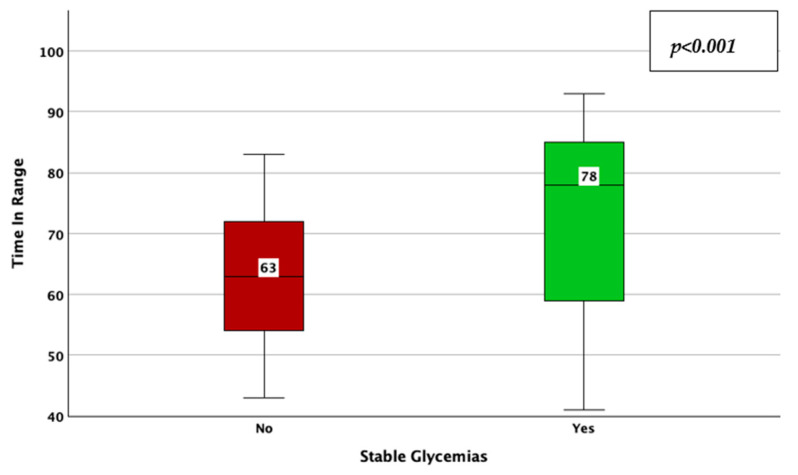
Time in range in patients with vs. without stable glycemic values, as described by a coefficient of variation lower than 36%.

**Figure 6:**
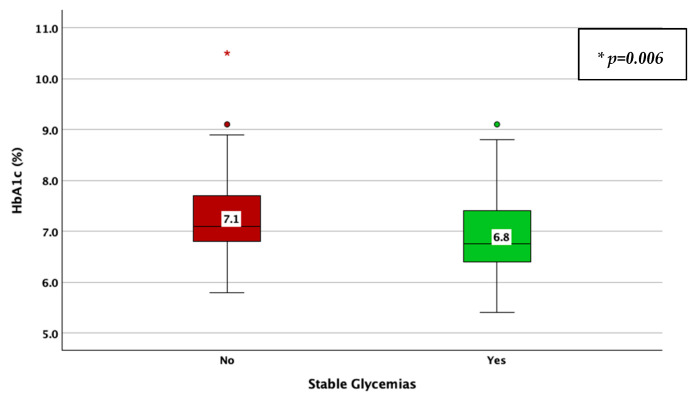
HbA1c in patients with vs. without stable glycemic values, as described by a coefficient of variation lower than 36%.

**Figure 7:**
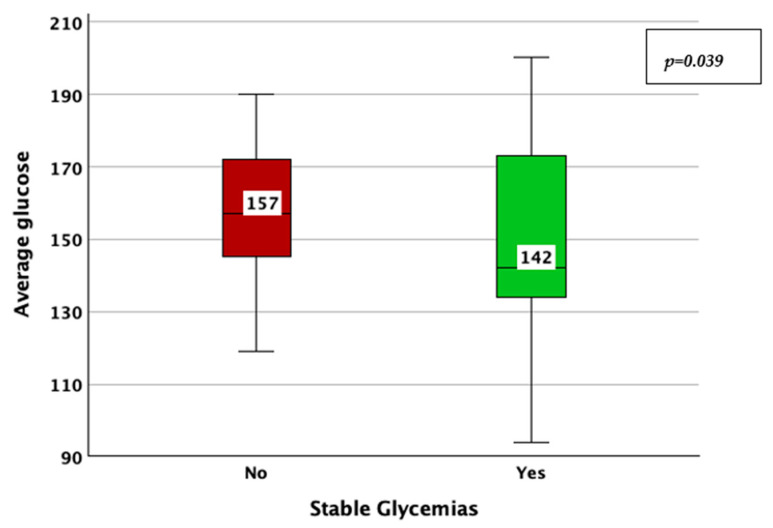
Average blood glucose in patients with vs. without stable glycemic values, as described by a coefficient of variation lower than 36%.

**Figure 8:**
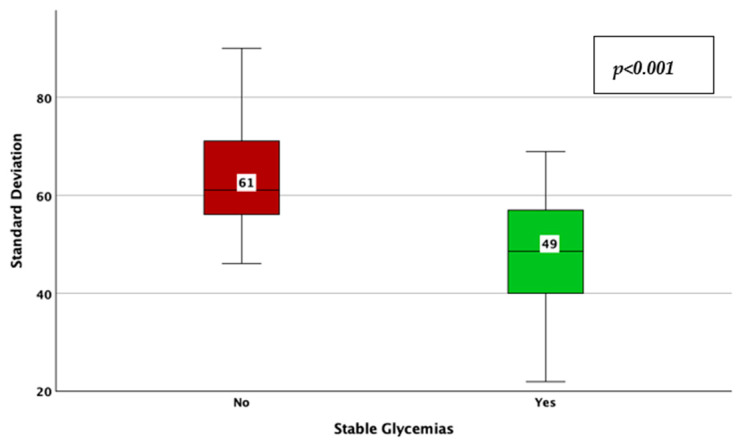
Standard deviation of blood glucose in patients with vs. without stable glycemic values, as described by a coefficient of variation lower than 36%.

The achievement of stable glycemic values, as defined by a CV < 0.36, was associated with a higher TIR (78% vs. 63%; *p* < 0.001; adjusted *p*-value < 0.001; Mann–Whitney U test; Figure 5), a lower HbA1c (6.8% vs. 7.1%; *p* = 0.006; adjusted *p*-value = 0.03; Mann–Whitney U test; Figure 6), average glucose (142 mg/dL vs. 157 mg/dL; *p* = 0.039; adjusted *p*-value = 0.195; Mann–Whitney U test; Figure 7), and SD of BG (49 mg/dL vs. 61 mg/dL; *p* < 0.001; adjusted *p*-value < 0.001; Mann–Whitney U test; Figure 8), while the association with diabetes duration was not statistically significant (*p* = 0.196; adjusted *p*-value = 0.98; Mann–Whitney U test).

**Figure 9:**
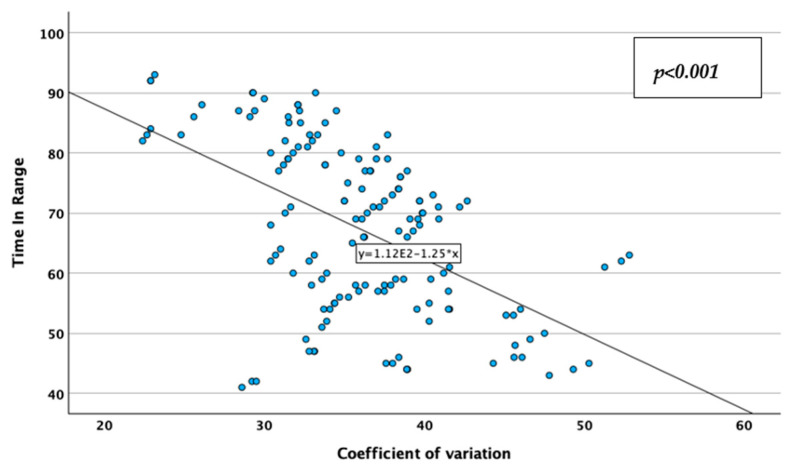
Correlations between coefficient of variation and time in range.

**Figure 10:**
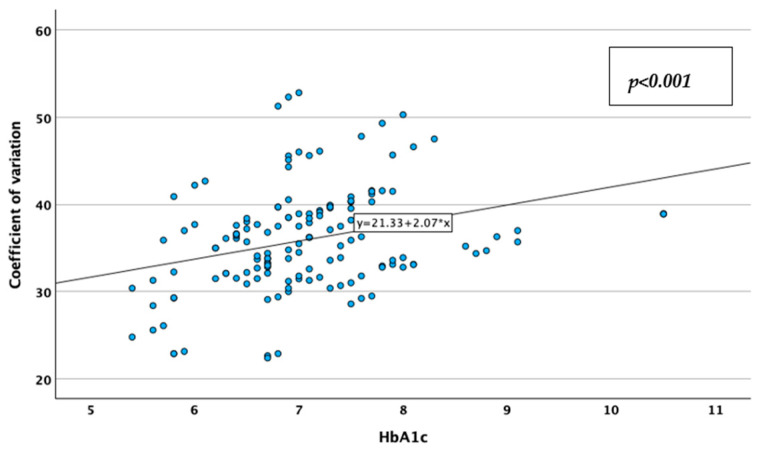
Correlation between HbA1c and coefficient of variation of blood glucose.

**Figure 11:**
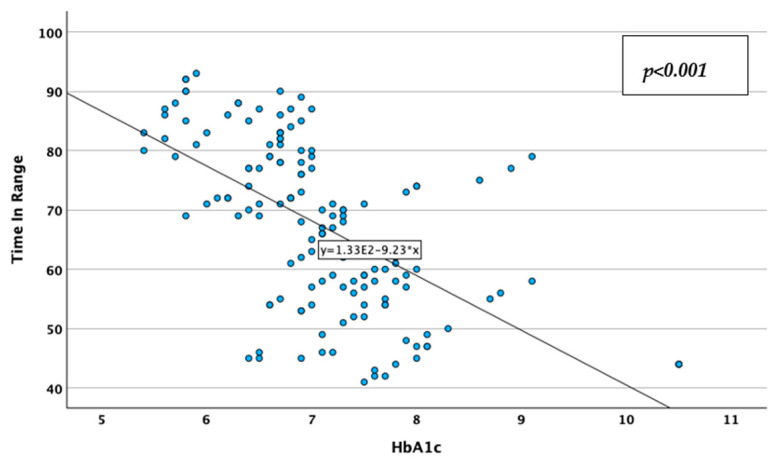
Correlations between HbA1c and time in range.

CV was reversely, moderately, and statistically significantly correlated with the TIR (Spearman’s r = −0.513; *p* < 0.001; Figure 9), and positively, moderately, and statistically significantly correlated with the HbA1c value (Spearman’s r = 0.349; *p* < 0.001; Figure 10), while TIR was reversely, moderately, and statistically significantly correlated with HbA1c (Spearman’s r = −0.637; *p* < 0.001; Figure 11). These associations lead to the observations that glycemic instability increases in patients with higher HbA1c levels and decreases in patients with more glycemic values in the desired range, while patients tend to stay in the desired range of glycemic values for less time when they have higher HbA1c values. Correlations data are represented in Table 3.

**Figure 12:**
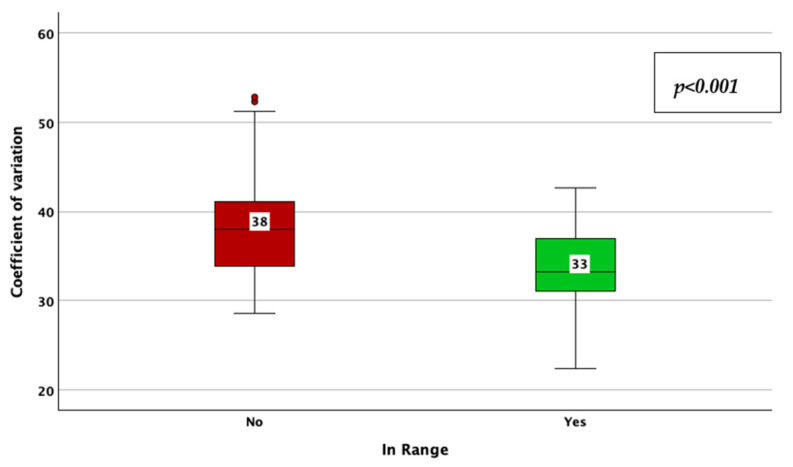
Coefficient of variation of blood glucose in patients achieving vs. not achieving the time in range target (more than 70%).

**Figure 13:**
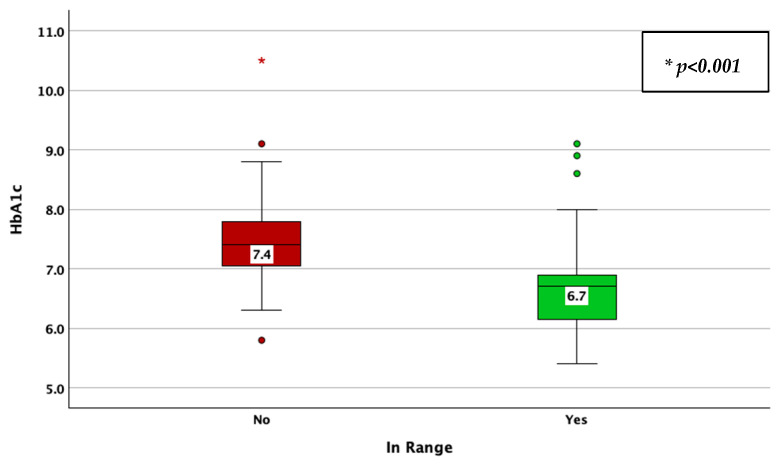
HbA1c in patients achieving vs. not achieving the time in range target (more than 70%).

**Figure 14:**
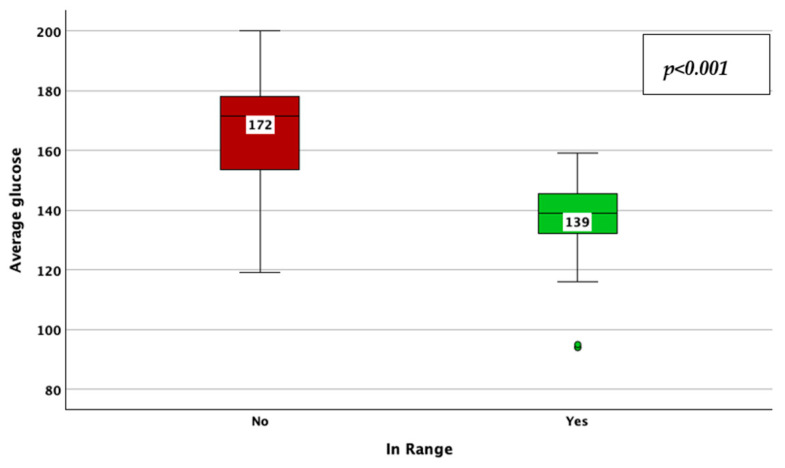
Average blood glucose in patients achieving vs. not achieving the time in range target (more than 70%).

**Figure 15:**
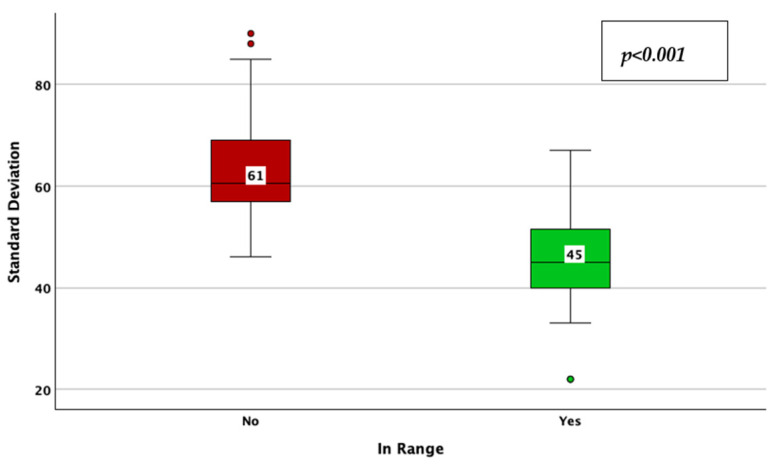
Standard deviation of blood glucose in patients achieving vs. not achieving the time in range target (more than 70%).

The achievement of TIR therapeutic targets, as defined by a TIR > 70%, was associated with a lower CV (33% vs. 38%; *p* < 0.001; adjusted *p*-value < 0.001; Mann–Whitney U test; Figure 12), HbA1c (6.7% vs. 7.4%; *p* < 0.001; adjusted *p*-value < 0.001; Mann–Whitney U test; Figure 13), average glucose (139 mg/dL vs. 172 mg/dL; *p* < 0.001; adjusted *p*-value < 0.001; Mann–Whitney U test; Figure 14), and SD of BG (45 mg/dL vs. 61 mg/dL; *p* < 0.001; adjusted *p*-value < 0.001; Mann–Whitney U test; Figure 15), while the association with diabetes duration was not statistically significant (*p* = 0.175; adjusted *p*-value = 0.875; Mann–Whitney U test).

The multivariate, forward-stepwise, conditional, logistic regression model built with the achievement of stable glycemic values, as described by a CV < 0.36, constructed as per the stepwise algorithm (inclusion criteria *p* < 0.05; exclusion criteria *p* > 0.15 per each individual evaluated possible predictor), included as valid predictors the average glucose measured by the sensor and TIR, while it excluded HbA1c and diabetes duration. This model proved to be a good fit for the biological phenomena (Nagelkerke’s R^2^ = 0.208; *p* < 0.001), meaning that the variation in the two selected predictors explains 20.8% of the variation in the probability of achieving stable glycemic values. As per the regression’s equation, for each increase of 1 mg/dL in the average glucose measured, the probability of having unstable glycemic values increased by 3.5% (Exp(ß) = 1.035 (95%CI: 1.004–1.067; *p* = 0.026)), while for each increase of 1% in the time spent in the desired range the probability of having stable glycemic values increased by 10.6% (Exp(ß) = 1.106 (95%CI: 1.051–1.163; *p* < 0.001)).

The multivariate, forward-stepwise, conditional, logistic regression model built with the achievement of a recommended time spent in the desired glycemic range, as described by a TIR > 70%, constructed as per the stepwise algorithm (inclusion criteria *p* < 0.05; exclusion criteria *p* > 0.15 per each individual evaluated possible predictor), included as valid predictors the average glucose values and the CV. This model is a good fit for the biological phenomena (Nagelkerke’s R^2^ = 0.646; *p* < 0.001), meaning that the variation in the two selected predictors explains 64.6% of the variation in the probability to achieve a TIR > 70%. As per the regression’s equation, for each increase of 1 mg/dL in the average glucose measured the probability of having a TIR > 70% decreased by 9.4% (Exp(ß) = 0.906 (95%CI: 0.876–0.936; *p* < 0.001)), while for each increase of 1 point in the CV, the probability of having a TIR > 70% decreased by 19.3% (Exp(ß) = 0.807 (95%CI: 0.725–0.898; *p* < 0.001)).

## 4. Discussions

The present study provides valuable insights into GV and its relationship with key factors that influence GV in T1DM patients, highlighting the complexity of diabetes management in achieving stable glycemic control.

While the associations between GV, TIR, and HbA1c have been previously reported, our study contributes novel insights by examining these relationships through the lens of composite glycemic targets and identifying their frequency in a real-world T1DM cohort. The incorporation of CGM-derived GV metrics into predictive models also offers practical relevance, despite moderate statistical power, by identifying which metrics are most closely associated with optimal glycemic profiles.

The baseline characteristics of patients included in the study regarding glycemic control indicated that the cohort was at the borderline of adequate diabetes management. While not fully optimized, the central tendency markers of glycemic profiles of these individuals revealed a median CV of less than 36%, an HbA1c at the threshold value of 7%, and a TIR that was 1% lower than the target of 70%.

Results of the study demonstrated that obtaining and maintaining stable glycemic values, as indicated by a CV < 0.36, is strongly associated with improvements in overall glycemic control. This is evidenced by a significantly higher TIR, lower SD, and lower average of glycemic values among patients who achieved the targeted CV, compared to those who did not reach this target. These findings emphasize the critical importance of CV as a robust and comprehensive marker of glucose stability, which can contribute to the prevention of heightened risk for both hyperglycemia and hypoglycemia, conditions that can have serious consequences for individuals with T1DM.

Furthermore, the study findings revealed a moderate but significant inverse correlation between TIR and HbA1c. This supports the notion that increased time spent within the optimal glycemic range correlates with lower HbA1c levels, highlighting the complementary nature of these two metrics, with TIR providing insight into daily glucose fluctuations that may not be fully captured by HbA1c alone.

Additionally, the results indicate a nuanced relationship between HbA1c and GV. The observed positive correlation between HbA1c and CV indicates that higher HbA1c levels are associated with higher CV, therefore, with higher glycemic instability. This further suggests that while HbA1c remains an important indicator of long-term glycemic control, it does not fully capture the complexities of daily glucose fluctuations, therefore, GV parameters can provide additional clinically relevant information on BG levels.

From a clinical perspective, the study results emphasize the need to incorporate GV metrics into routine diabetes management. While HbA1c remains a gold-standard marker for long-term control, focusing on achieving an optimal level of TIR and CV may help clinicians better predict and manage glucose fluctuations, ultimately reducing the risk of diabetes-related complications [39].

The multivariate logistic regression analysis revealed that achieving stable glycemic values is significantly influenced by average glucose and TIR, while diabetes duration and HbA1c were not significant predictors, suggesting that real-time glucose monitoring metrics may provide a more accurate assessment of GV than traditional measurements alone. Additionally, the model in the study for predicting TIR > 70% identified average glucose and CV as key predictors, explaining 64.6% of the variation in TIR achievement. These findings reinforce the clinical utility of CGMSs in guiding individualized treatment adjustments.

The strengths of this study are represented by the large size of the study cohort, which included participants from two major diabetes centers in Romania. All patients utilized continuous glucose monitoring system devices from the same manufacturer, ensuring consistency and validity of the measured parameters. Furthermore, the high adherence rate of over 80% time adherence to the CGMS protocol further bolsters the validity of the collected data. The data collection process was rigorous, and the laboratory tests were performed at certified medical laboratories, enhancing the overall quality and reliability of the findings.

Several important limitations of this study should be acknowledged. First, the observational design precludes causal inference. Second, the logistic regression model predicting GV had limited explanatory power (R^2^ = 0.208), indicating that key predictors may be missing. Although we evaluated clinical variables such as age, diabetes duration, and insulin therapy type, these did not retain statistical significance in the final models. Future studies should aim to include a broader array of clinical and behavioral variables—such as hypoglycemia unawareness, physical activity, meal patterns, and psychosocial factors—to enhance model robustness. Nonetheless, these limitations do not detract from the valuable insights gained from this study, which contributes to the growing body of evidence on the clinical relevance of GV in the management of T1DM.

Although Bonferroni correction is conservative and may increase the risk of type II error, we chose this approach to reduce the chance of false positives and ensure robustness of our findings across multiple comparisons.

The study findings align with previous research that underscores the significance of GV in predicting glycemic control and microvascular diabetes complications. Prior research has demonstrated that reducing GV can decrease the risk of dysglycemic events and improve long-term metabolic outcomes [40,41,42].

A study involving 515 patients with T1DM who utilized CGMSs along with insulin pumps revealed that lower TIR values were associated with an increased risk of developing microvascular complications related to diabetes, as well as a higher frequency of hospital admissions due to acute diabetes-related complications, such as diabetic ketoacidosis and severe hypoglycemia [43].

Results of various studies also suggest that focusing solely on achieving an optimal HbA1c level may not be sufficient for preventing diabetes complications [44,45,46,47]. A study that included 32 patients with T1DM divided into two groups based on the presence of microvascular complications revealed that the group with microvascular complications had a higher CV and higher SD in their glycemic values, despite not having significant differences in their HbA1c levels compared to those without microvascular complications. This highlights the importance of considering additional markers of glycemic control, beyond just HbA1c, as they can provide valuable insights into the overall stability and variability of BG levels [48].

Another study revealed that in critically ill patients, higher GV expressed as higher CV was shown to be an independent risk factor for cognitive impairments and mortality [49].

Additionally, emerging evidence suggests that GV may play a significant independent role in the development and progression of macrovascular complications in T1DM, particularly CVEs. Diabetes mellitus is already a well-established risk factor for CVD, with diabetic patients having up to a 4-fold increased risk of adverse cardiovascular outcomes compared to the general population. However, recent studies indicate that the degree of GV experienced by these patients may confer an additional, independent CVR. Specifically, higher levels of GV have been observed in patients with acute coronary syndromes, atherosclerotic vascular disease, cerebrovascular disease, and other major CVEs [50,51,52,53,54].

The mechanisms by which glycemic fluctuations contribute to these microvascular and macrovascular complications are an area of active investigation as a deeper understanding of this relationship could inform the development of more targeted treatment strategies to reduce the risk of comorbidities associated with T1DM. Currently, the literature data suggests that the increased GV observed in T1DM can contribute to the development of oxidative stress and endothelial dysfunction. Rapid fluctuations in BG levels lead to excessive mitochondrial activity, resulting in an elevated production of reactive oxygen species (ROS). Moreover, hypoglycemic events associated with higher GV are activating the sympathetic nervous system, leading to a surge in adrenaline and noradrenaline that stimulate NADPH oxidase, further increasing ROS production, particularly in blood vessels and the heart. This imbalance between ROS generation and antioxidant defenses can result in oxidative damage to cellular components, impairing endothelial function and potentially contributing to the increased risk of vascular complications observed in individuals with T1DM and a high GV [55,56,57,58,59,60].

Another potential mechanism associated with GV is increased platelet aggregation. Higher GV has been consistently linked to increased platelet activation and aggregation. This heightened platelet reactivity can promote the development of thrombosis, which in turn can lead to vascular occlusion in various territories, including the microvasculature. Such vascular complications can exacerbate the progression of diabetic nephropathy and retinopathy, as well as increasing the risk of cardiovascular events.

The presence and prognosis of diabetic neuropathy has also been linked to higher GV. The underlying mechanisms are complex and not yet fully understood, but GV is thought to contribute to the development of diabetic nephropathy and diabetic peripheral neuropathy through various pathways.

These include oxidative stress, mitochondrial dysfunction, advanced glycation end-products, and dysregulated metabolic pathways, along with Schwann cell dysfunction, due to various processes such as apoptosis, lipid abnormalities, and inflammation. The precise mechanisms by which GV influences the development and progression of these diabetic complications are still being actively investigated, but the available evidence suggests that GV is an important factor to consider in the comprehensive management of T1DM [61,62].

Future research should explore the impact of targeted GV interventions on long-term diabetes outcomes. Prospective interventional studies are needed to assess whether optimizing CV and TIR can lead to sustained improvements in metabolic control and reduced complication risks. Additionally, the integration of advanced CGM-based algorithms for predicting GV and adjusting insulin therapy warrants further investigation.

## 5. Conclusions

Stable glycemia, as defined by a CV lower than 36%, was associated with a significantly lower HbA1c, and average and SD of blood glucose, and a significantly higher TIR. Achievement of the recommended TIR (higher than 70% of the monitored time) was associated with a significantly lower HbA1c, and SD and average blood glucose.

Only 28.6% of the patients with T1DM achieved the composite GV and metabolic outcome, as defined by achieving at the same time a TIR > 70%, CV < 0.36, as well as a HbA1c < 7%. However, 53.7% of them achieved the HbA1c target, the traditional indicator of the quality of glycemic control; this emphasize the crucial role of evaluating the GV in the overall assessment of the quality of glycemic control, alongside the traditional biomarkers like HbA1c or blood glucose levels. It also emphasizes the need for a comprehensive approach to diabetes care that extends beyond the traditional reliance on HbA1c and instead incorporates CGMS-derived GV metrics as essential targets for optimizing personalized treatment strategies.

## Figures and Tables

**Table 1:** Patient’s baseline characteristics.

Parameter		Result
Gender ^a^	Men	60 (40.8%)
	Women	87 (59.2%)
Age ^b^		32 years [15]
Diabetes Duration ^b^		7 years [10]
HbA1c ^b^		7.0% [0.9]
Therapy type ^a^	Basal bolus (multiple injections)	84 (57.1%)
	Insulin pump	63 (42.9%)

^a^ Categorical variables. Results are presented as absolute frequencies and (percentage from the entire group). ^b^ Continuous variables with non-parametric distribution. Results are presented as median and [interquartile distance].

**Table 2:** Central tendency and dispersion analysis of glycemic variability indicators.

	Median	95.0% Lower CL for Median	95.0% Upper CL for Median	Percentile 25	Percentile 75	Minimum	Maximum
Standard Deviation (mg/dL)	57	56	59	46	63	22	90
Coefficient of variation	35.9	34.5	37.0	32.1	39.1	22.4	52.8
Time In Range (%)	69	66	72	56	79	41	93
HbA1c (%)	7.0	6.9	7.2	6.6	7.5	5.4	10.5

Values are presented as medians with 95% confidence intervals, interquartile ranges (25th and 75th percentiles), and observed minimum/maximum values.

**Table 3:** Correlation matrix between coefficient of correlation of blood glucose, time in range, and HbA1c.

Correlations			
		Coefficient of Variation	Time in Range
Time in Range	Spearman’s r	−0.513 **	--
	*p*-value	<0.001	.
	*n*	147	147
HbA1c	Spearman’s r	0.349 **	−0.637 **
	*p*-value	<0.001	0.000
	*n*	147	147

** Correlation is significant at the 0.01 level (2-tailed).

## Data Availability

The data presented in this study is available on request from the corresponding author. The data are not publicly available due to local privacy and data protection regulations.

## Abbreviations

The following abbreviations are used in this manuscript:

GV	Glycemic variability
TIR	Time in range
SD	Standard deviation
T1DM	Type 1 diabetes mellitus
BG	Blood glucose
HbA1c	Hemoglobin A1c
CGMS	Continuous glucose monitor system
CV	Coefficient of variability
T2DM	Type 2 diabetes management
CVD	Cardiovascular disease
CVR	Cardiovascular risk
CVE	Cardiovascular event
